# On Medicinal Cigars

**Published:** 1852-01

**Authors:** 


### *On Medicinal Cigars.* By Dr. Landerer.

The employment of various organic and inorganic substances of a volatilisable nature in a cigar form, has frequently been resorted to. In this way, stramonium, cicuta, Raspail's camphor, and corrosive sublimate have been used by means of tobacco deprived of its nicotin. The great efficacy of this last substance in ulcerated syphilitic throat, in Dr. Landerer's hands, has rendered him very desirous of extending this form of medication. He prepared cigars, therefore, by moistening tobacco freed from nicotin with tinct. of iodine, a solution of iodide of mercury in sulphuric æther, or a solution of iodide of potassium. He found these cigars of great utility in syphilitic ulceration of the throat, and in ozæna. So, too, by moistening the tobacco with an ætherial solution of hyoscyamin, he has relieved most obstinate spasmodic cough without inducing any narcotism. Among other substances tried, he found a solution of creosote in spirit of wine and æther, a very useful form in scorbutic ulceration of the gums. Cigars moistened with *tinct. moschi* relieved hysterical and spasmodic coughs; and a case of severe hysterical paroxysms, occurring in an irritable subject, was advantageously treated by the alcoholic solution of the acetate of morphia. Cigars formed of this substance are also very useful in the toothache. Arsenical cigars, formed by steeping the tobacco in Fowler's solution, have also been employed, and Dr. Landerer believes that this form of medication might be extended to a great variety of substances.—*Buchner's Repert.*, B. vi, p. 347.

